# A Comparative Study to Evaluate the Safety and Efficacy of Microneedling as a Stand-Alone Treatment for Striae Rubrae and Albae

**DOI:** 10.1093/asj/sjaf261

**Published:** 2025-12-17

**Authors:** Simona Marin, Abigail Watterson, Mona L Alqam, Brian C Jones, Thomas M Hitchcock

## Abstract

**Background:**

Striae distensae are a common and often emotionally distressing dermatologic condition among adults. While therapeutic modalities are available, none completely resolve the visual or morphological changes of stretch marks.

**Objectives:**

This study sought to evaluate the safety and efficacy of microneedling as a stand-alone treatment for both immature (striae rubrae) and mature (striae albae) stretch marks.

**Methods:**

Fifteen striae rubrae and 19 striae albae regions from 29 subjects received 4 microneedling treatments, spaced monthly. Site-matched regions on opposite sides of the body were evaluated as untreated controls. Manchester Scar Scale (MSS) and Clinician's Global Aesthetic Improvement Assessment Scale (CGAIS) assessments were completed at all visits including follow-up at 3- and 6-months post-last microneedling treatment. Secondary endpoints included length measurements, adverse event monitoring, safety assessments, post-procedure symptom severity, and subject satisfaction.

**Results:**

Both striae rubrae and striae albae showed progressive improvements in clinical assessments across visits, including continued improvement between 3- and 6-months posttreatment. When comparing groups, striae rubrae had superior aesthetic outcomes, including 43.89% and 48.89% MSS score improvements over baseline at 3 and 6 months, respectively, compared with 36.69% and 41.61% for striae albae. While post-procedure reactions were also heightened for striae rubrae, all reactions were transient, and no adverse events were reported.

**Conclusions:**

This study supports microneedling as a safe and effective treatment for striae rubrae and striae albae, offering preliminary evidence that intervening with microneedling in early-stage stretch marks (striae rubrae) can yield better cosmetic outcomes compared to mature stretch marks (striae albae).

**Level of Evidence 3 (Therapeutic):**

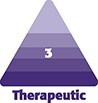

Striae distensae, or stretch marks, are linear dermal scars that arise from mechanical stretching of the skin, creating a disruption of the collagen and elastin networks.^1^ Affecting both men and women, striae frequently occur with puberty, rapid weight fluctuations, corticosteroid use and, most commonly, pregnancy, impacting as many as 90% of pregnant women.^2,3^ Clinically, striae distensae are categorized into 2 stages: *striae rubrae*, the acute form characterized by erythematous and slightly raised lesions, and *striae albae*, the chronic form marked by hypopigmented, atrophic lines that often persist long term.^1,4,5^ While rarely detrimental to one's physical health, striae can considerably affect the quality of life and self-esteem of those affected.^6^

A variety of therapeutic modalities have been attempted to treat striae distensae, including topical agents (eg, tretinoin, hyaluronic acid, laser and light devices such as fractional CO₂ and pulsed dye lasers, chemical peels, microdermabrasion, platelet-rich plasma (PRP), and radiofrequency treatments).^7-17^ However, no current treatments fully correct striae morphology and appearance, and many interventions have limited evidence or are associated with high costs, risk of adverse effects (especially in patients with darker skin types), or require multiple modalities to achieve improvement. Thus, striae distensae persists as one of the most common yet challenging dermatologic conditions.^2,12,13^

Microneedling has emerged as a promising minimally invasive technique which induces controlled dermal injury, triggering a wound healing response coupled with increased collagen and elastin production.^18^ Several studies have shown that proper microneedling techniques with automated, medical-grade devices can improve skin texture, elasticity, and pigmentation, making it an appealing treatment for atrophic scars and photoaged skin.^19-25^ While microneedling has been investigated as a potential treatment modality for striae distensae, the current literature describes variable treatment approaches and outcomes, and several questions remain regarding the utility of microneedling as a stand-alone treatment for striae rubrae and striae albae specifically.^12,26-29^ Moreover, few studies have assessed long-term outcomes or directly compared treatment responses between striae types,^30^ thus demonstrating a need for establishing an optimized treatment strategy and intervention timeline for producing optimal outcomes.

This prospective clinical study was designed to evaluate the safety and efficacy of microneedling as a stand-alone therapy for both immature (striae rubrae) and mature (striae albae) stretch marks. By incorporating reputable clinical scales, photographic documentation, and follow-up assessments at 3 and 6 months post-treatment, this investigation aimed to clarify the therapeutic potential of microneedling in the management of striae rubrae and striae albae and to examine potential differences in treatment responses between lesion subtypes.

## METHODS

### Participants

A total of 36 healthy adults (33 female, 3 male) aged 18-65 with clinically diagnosed striae rubrae and/or striae albae (present for more than 6 weeks) were enrolled in the study, which lasted from May 2023 to April 2024. All subjects met and adhered to predefined inclusion and exclusion criteria, which included being in generally good health, willing to maintain stable body weight and exercise levels throughout the study, and discontinuing any other stretch mark therapies. Women of childbearing potential agreed to use acceptable contraception throughout the study.

Key exclusion criteria included nursing, pregnancy or plans to become pregnant, known allergies to topical lidocaine or skincare products, history of keloid or hypertrophic scarring, active skin disease, immunosuppressive conditions, systemic illness affecting wound healing, recent or planned treatment in the stretch mark area, and uncontrolled medical conditions. Subjects were also excluded if they had recent cosmetic procedures (eg, lasers, microneedling, radiofrequency, PRP, etc.), were on medications such as systemic corticosteroids, anticoagulants or topical retinoids, or were currently enrolled in another investigational study. Individuals with any condition deemed by the investigator to interfere with study participation or outcomes were excluded.

### Study Design

This prospective, open-label clinical study sought to evaluate the safety and efficacy of microneedling as a stand-alone treatment for striae distensae, including both striae rubrae and striae albae. The study adhered to ethical principles expected for a clinical study and was approved by the relevant Institutional Review Board (IRB; Allendale IRB, 30 Neck Road Old Lyme, Connecticut, 06371; study number CL-SM-23-03). All participants provided written informed consent and photography release agreement prior to enrollment.

The total study duration per subject was 9 months, during which participants attended 6 clinic visits: Baseline (day 1), Visit 2 (week 4), Visit 3 (week 8), Visit 4 (week 12), Visit 5 (week 24), and Visit 6 (final visit; week 36). Microneedling treatments were administered at Visits 1 through 4, during which each subject had one or 2 stretch mark regions treated based on clinical judgment and subject eligibility. Follow-up assessments were completed 3 months (Visit 5) and 6 months (Visit 6) following the last microneedling treatment.

Treatment regions were selected at the initial visit and assigned as either striae albae or striae rubrae according to their color and characteristics. Selected treatment regions received 4 microneedling sessions, spaced 4 weeks apart, using one of 2 FDA-cleared devices. Both microneedling devices (SkinPen Precision® and MicroPen EVO™; Crown Laboratories, Inc., Johnson City, TN) are classified as Class II surgical instruments under CFR 878.4430 and use a motorized active retraction mechanism which delivers accurate positive extension and retraction of 14 32-guage needles within the skin. Treatment areas included the abdomen, hips, and shoulders. Stretch marks in the same anatomical location on the contralateral side of the body were assessed as untreated control sites.

### Microneedling Treatments

Microneedling treatments were administered by a designated trained professional in adherence with the device Instructions for Use (IFU). Prior to treatment, a prescription-grade numbing cream was applied to the treatment area(s) for at least 20-30 minutes. During the procedure, a topical hydrogel was applied to the skin to reduce friction and minimize epidermal trauma. The appearance of even-level of erythema was considered an adequate treatment response and represented the primary clinical endpoint. The presence of pinpoint bleeding was also recognized as a clinical endpoint.

Each microneedling session involved 3 passes with adjusted treatment depths based on skin condition, anatomical location, and patient tolerance, as determined by the study investigator. Treated regions were comprised of both the selected stretch marks and the immediately surrounding tissue. Treatment depths ranged from 0.25 to 2.5 mm and generally increased in depth with each subsequent visit (Supplemental Table 1). No significant differences in treatment depths were observed between the striae rubrae and striae albae subgroups (Supplemental Table 1).

### Study Visits and Evaluations

Clinic visits were conducted at Baseline (Day 1), during which participants underwent informed consent, baseline assessments and Treatment 1, Week 4 (Treatment 2), Week 8 (Treatment 3), Week 12 (Treatment 4), Week 24 (3-month follow-up), and Week 36 (6-month follow-up). Stretch mark regions that met the inclusion criteria and were apparent on both the sinistral and dextral sides of the body were selected during the initial visit and classified as either striae rubrae or striae albae based on color and characteristics. Corresponding left and right regions were then randomly assigned to treatment or control groups using a computer-simulated coin toss.

Safety and efficacy assessments were completed at each treatment visit (Visits 1-4), and subjects returned for follow-up assessments roughly 3 and 6 months following the final microneedling treatment (Visits 5 and 6). Assessments at each visit included stretch mark length measurements, in which the longest stretch mark was measured, and standard digital photography for both the treated and untreated control sites. Manchester Scar Scale (MSS), Clinician's Global Aesthetic Improvement Scale (CGAIS), and subject questionnaires were completed for the treated region(s) at every visit. Investigator's assessment of post-microneedling safety parameters (erythema, edema, burning/stinging, dryness, itching, scaling/peeling) were scored by the study investigator immediately following each microneedling treatment (Visits 1-4). In the weeks following each procedure, subject-reported symptom severity was rated daily using a 4-point grading scale (0 = none, 1 = mild, 2 = moderate, 3 = severe). Subject satisfaction questionnaires were completed at Visits 4-6, and responses were later converted to numerical values from 1 (strongly disagree) to 5 (strongly agree) for graphical representation. Adverse events were monitored throughout the study.

### Photographic Assessments and Blinded Grading

Standardized digital photographs were obtained at all visits for treated and site-matched control regions. Images were captured before and after microneedling, and representative photographs from each treatment group were selected for figure preparation. Although image magnification varied among subjects to account for differences in stretch mark size, magnification settings and image editing for enhanced visibility (if any) were applied consistently across visits and site-matched regions for each subject.

Following study completion, sets of unlabeled images from baseline and 6-month follow-up for all 34 treatment sites and their respective untreated control regions were randomized using a random number generator and graded by 3 independent clinical experts based on observations between photos “A” and “B”. Graders, who were blinded to the visit and treatment status, compared each set of photos using a modified CGAIS-style scale. For each set, graders first identified which image represented baseline and follow-up based on their judgment, then rated the degree of improvement over “baseline” on a 0-3 scale (0 = no change, 1 = improved, 2 = much improved, 3 = very much improved, ie, optimal cosmetic outcome). Ratings in which graders incorrectly identified baseline and follow-up yet scored an improvement (≥1) were adjusted to negative values for final analysis.

### Statistical Analysis

Safety and efficacy outcomes were analyzed for the 34 total treatment regions that completed the study. Data from discontinued subjects (*n* = 7 regions) were excluded from the final analysis. Descriptive statistics were used to summarize demographic and clinical characteristics for each striae type. To calculate percent change over baseline, data from each striae region were normalized to their respective baseline values and subsequently pooled by group and timepoint. For all scale-based outcomes (eg, MSS, CGAIS, length measurements, subject-reported symptom severity), mean values (±standard deviation [SD]) were compared across timepoints and striae subgroups using 2-way ANOVA with Tukey's multiple comparisons in GraphPad Prism 10 (v10.4.2). Statistical significance was defined as *P* < .05. Significant differences between timepoints within a subgroup are denoted by asterisks (*P < .05, **P < .01, ***P < .001, ****P < .0001), and differences between groups are denoted by number signs (^#^*P* < .05, ^##^*P* < .01, ^###^*P* < .001, ^####^*P* < .0001). Secondary analyses were conducted to confirm that no considerable differences were observed between the 2 microneedling devices or based on subject demographics (data not shown).

## RESULTS

### Participant Demographics

A total of 41 treatment regions from 36 participants were enrolled in the study. Of those enrolled, 29 subjects completed the study, collectively contributing 34 total treatment regions (15 striae rubrae and 19 striae albae) to the final analysis. Five of the 7 discontinued subjects withdrew consent for reasons unrelated to the treatment protocol, and the remaining 2 were lost to follow-up.

Study participants received 4 monthly microneedling sessions, followed by follow-up visits approximately 3 and 6 months after the final treatment, occurring on average at 87.2 days (range, 72-98) and 209.6 days (range, 182-233), respectively. Participant ages ranged from 18 to 65, with the mean participant age at enrollment being 38.1 years (Table 1). Of the 34 completed treatment regions, 29 (85.3%) were from female participants and 5 (14.7%) were from males. Fitzpatrick skin types (FST) ranged from I through VI, with types IV (32.4%) and II (23.5%) being the most common (Table 1). Skin types and anatomical locations of the stretch marks were reasonably distributed between the 2 striae subtypes, though it should be noted that skin color affects the apparentness of the lesion subtypes and therefore did slightly influence the overall distribution of skin types between the 2 groups (Table 2). Nonetheless, analysis of treatment outcomes showed no statistically significant differences with respect to participant age, gender, skin type, race, or anatomical treatment location (data not shown). Additionally, while treatment depths varied on a subject-by-subject basis based on several patient- and site-specific parameters, no considerable correlations between depth of needle penetration and treatment outcomes were observed at any timepoint (data not shown).

**Table 1.** Summary of Demographics Across Subjects (*N* = 29) and Treatment Regions (*N* = 34)

	Subject demographics (*N* = 29)	Treatment site demographics (*N* = 34)
Age (at time of enrollment)		
Mean	38.1	37.6
Median	37	36
Range	18-65	18-65

	N	%	N	%
Sex				
Female	26	89.7%	29	85.3%
Male	3	10.3%	5	14.7%
Ethnicity				
Hispanic or Latino	7	24.1%	8	23.5%
Not Hispanic or Latino	22	75.9%	26	76.5%
Race				
White	17	58.6%	21	61.8%
African American	9	31.0%	9	26.5%
Asian	3	10.3%	4	11.7%
Other	0	0.0%	0	0%
Fitzpatrick skin type				
I	1	3.4%	1	2.9%
II	6	20.7%	8	23.5%
III	6	20.7%	6	17.6%
IV	8	27.6%	11	32.4%
V	3	10.3%	3	8.8%
VI	5	17.2%	5	14.7%

**Table 2.** Summary of Demographics and Treatment Site Locations by Group

Group	*N*	Treatment region			Mean age (range)	Fitzpatrick skin type					
		Abdomen	Hip	Shoulder/Arm		I	II	III	IV	V	VI
Striae Rubrae	15	9	4	2	31 (18-60)	0	4	5	6	0	0
Striae Albae	19	12	7	0	43 (18-65)	1	4	1	5	3	5

### Treatment Efficacy

**Figure 1.**
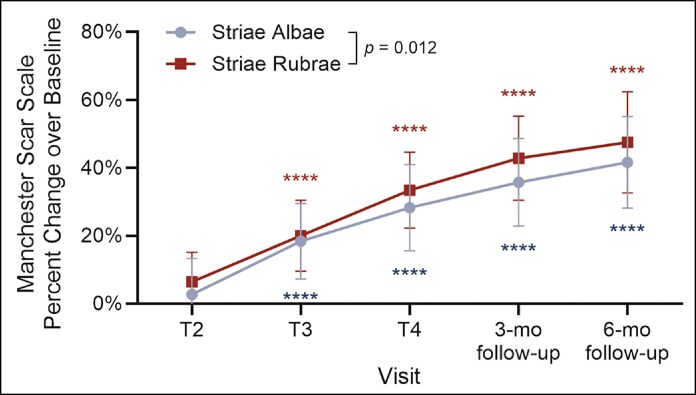
Relative Manchester Scar Scale (MSS) score improvement over baseline by stretch mark type and treatment visit. Shown are mean relative percent change over baseline ± SD for striae albae (blue; *n* = 19) and striae rubrae (red; *n* = 15) at treatment visits 2-4 (T2-T4) and follow-up visits 3- and 6-months post-last treatment. Asterisks correspond to the respective stretch mark type by color and denote significance (****P < .0001) compared with baseline. While no significant differences between striae albae and rubrae were observed at individual timepoints, striae rubrae demonstrated greater overall improvement compared to striae albae (*P* = .012) when compared as a whole.

Significant improvements in MSS assessment scores (based on color, finish, contour, discoloration, and texture) were observed across visits for both striae albae and striae rubrae, with progressive improvement seen from Visit 2 (Treatment 2; T2) through the 6-month follow-up (Figure 1). In evaluating outcomes between lesion subtypes, striae rubrae exhibited greater overall improvement than striae albae when compared collectively across visits (*P* = .012) (Figure 1).

**Figure 2.**
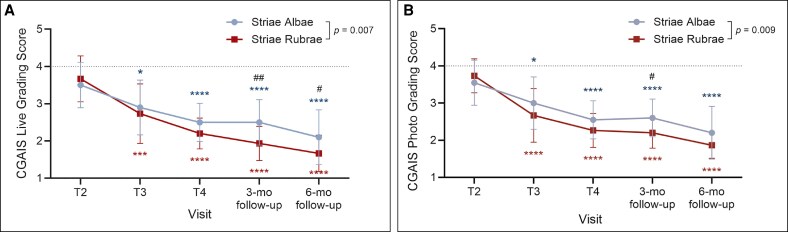
Clinician's Global Aesthetic Improvement Assessment (CGAIS) scores by stretch mark type and treatment visit. (A, B) Shown are mean CGAIS scores ± SD (lower scores = greater improvement) based on live grading (A) and photo grading (B) assessments for striae albae (blue; *n* = 19) and striae rubrae (red; *n* = 15). Dotted lines mark no change from baseline. Asterisks correspond to the respective stretch mark type by color and denote statistically significant improvement (*P < .05, ****P < .0001) compared to T2. Number signs indicate significance between groups (^#^*P* < .05).

Live and photographic assessments of treated regions using CGAIS also revealed significant (*P* < .0001) progressive improvements from the second treatment visit through the 6-month follow-up in both striae subtypes (Figure 2A, B). While considerable improvements were observed across the board, striae rubrae had consistently lower (ie, more favorable) CGAIS scores at follow-up, with significant differences between striae rubrae and albae observed at both follow-up visits (*P* < .05, *P* < .01). These findings suggest that treating striae in their acute, early-stage form (striae rubrae) may offer greater potential for superior aesthetic outcomes than mature lesions (striae albae).

Importantly, it is also worth noting that both groups showed continued improvement in both MSS and CGAIS assessments between the 3- and 6-month posttreatment timepoints (Figures 1 and 2). Although not statistically significant, this trend aligns with findings from previous microneedling studies^31^ and supports the idea that tissue remodeling continues for at least 3-6 months after completing a course of microneedling treatments.

**Figure 3.**
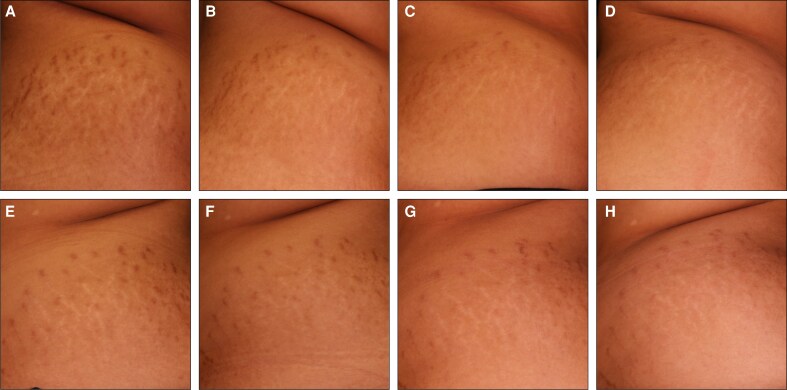
Site-matched treated and untreated striae rubrae on hips of 18-year-old female with FST IV. (A–D) Treated right hip at baseline (A), final treatment; T4 (B), 3 months post-last treatment (C), and 6 months post-last treatment (D). (E–H) Untreated left hip at baseline (E), final treatment; T4 (F), 3 months post-last treatment (G), and 6 months post-last treatment (H).

**Figure 4.**
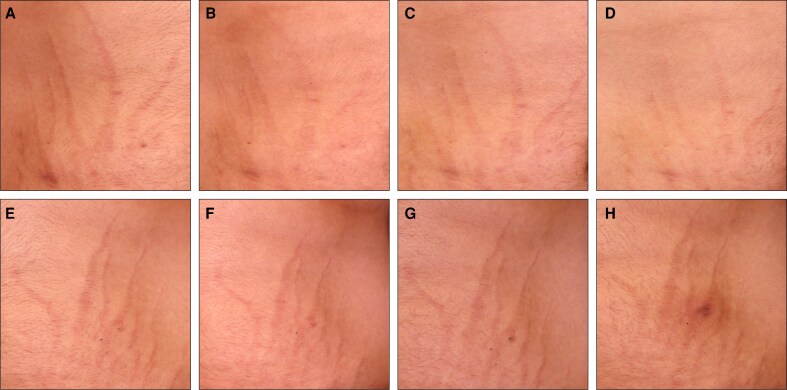
Site-matched treated and untreated striae rubrae on abdomen of 23-year-old male with FST IV. (A–D) Treated right abdomen at baseline (A), final treatment; T4 (B), 3 months post-last treatment (C), and 6 months post-last treatment (D). (E–H) Untreated left abdomen at baseline (E), final treatment; T4 (F), 3 months post-last treatment (G), and 6 months post-last treatment (H).

**Figure 5.**
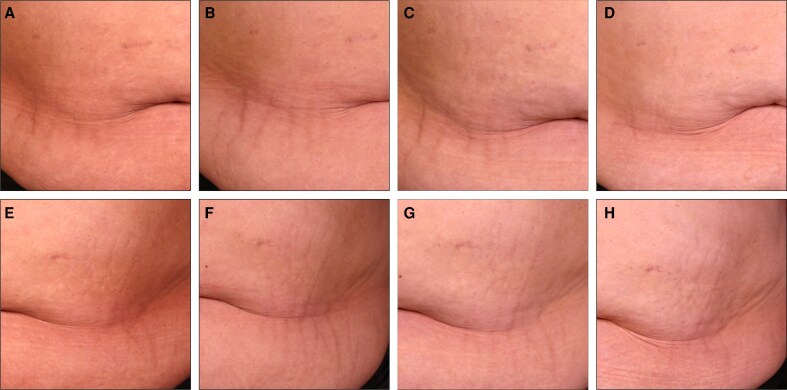
Site-matched treated and untreated striae rubrae on abdomen of 48-year-old female with FST IV. (A–D) Treated right abdomen at baseline (A), final treatment; T4 (B), 3 months post-last treatment (C), and 6 months post-last treatment (D). (E–H) Untreated left abdomen at baseline (E), final treatment; T4 (F), 3 months post-last treatment (G), and 6 months post-last treatment (H).

**Figure 6.**
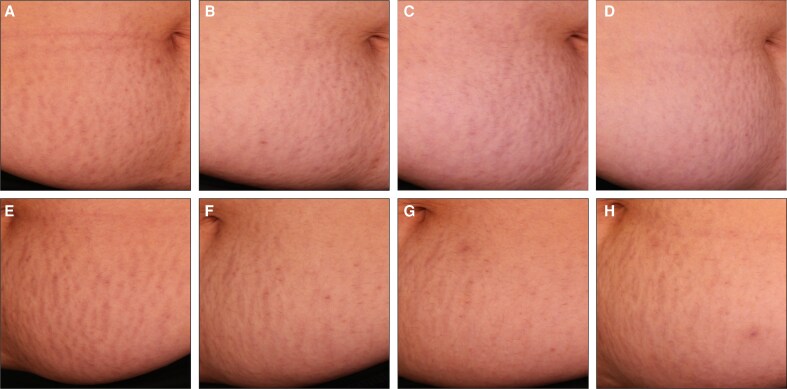
Site-matched treated and untreated striae rubrae on abdomen of 23-year-old female with FST III. (A–D) Treated right abdomen at baseline (A), final treatment; T4 (B), 3 months post-last treatment (C), and 6 months post-last treatment (D). (E–H) Untreated left abdomen at baseline (E), final treatment; T4 (F), 3 months post-last treatment (G), and 6 months post-last treatment (H).

**Figure 7.**
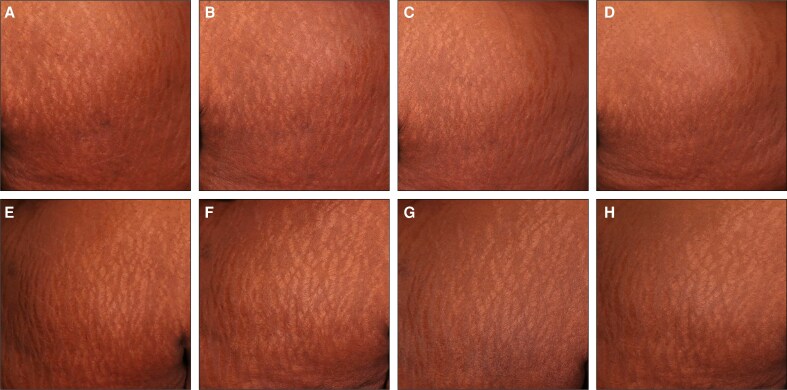
Site-matched treated and untreated striae albae on abdomen of 42-year-old female with FST VI. (A–D) Treated left abdomen at baseline (A), final treatment; T4 (B), 3 months post-last treatment (C), and 6 months post-last treatment (D). (E–H) Untreated right abdomen at baseline (E), final treatment; T4 (F), 3 months post-last treatment (G), and 6 months post-last treatment (H).

**Figure 8.**
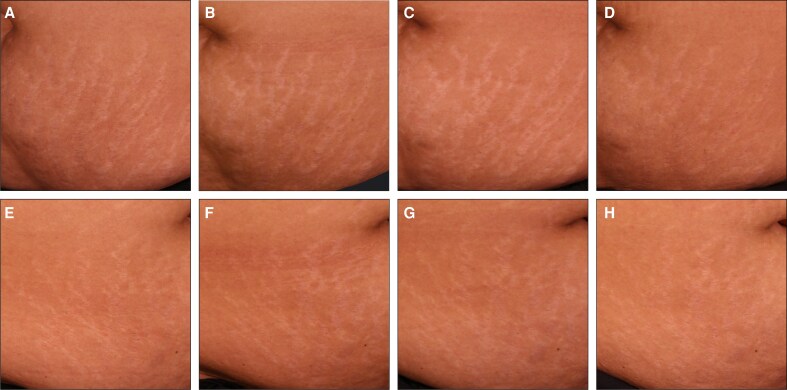
Site-matched treated and untreated striae albae on abdomen of 33-year-old female with FST IV. (A–D) Treated left abdomen at baseline (A), final treatment; T4 (B), 3 months post-last treatment (C), and 6 months post-last treatment (D). (E–H) Untreated right abdomen at baseline (E), final treatment; T4 (F), 3 months post-last treatment (G), and 6 months post-last treatment (H).

**Figure 9.**
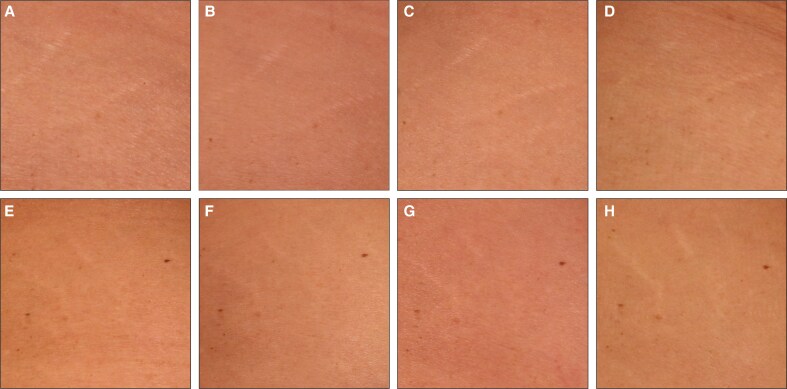
Site-matched treated and untreated striae albae on hips of 65-year-old female with FST III. (A–D) Treated right hip at baseline (A), final treatment; T4 (B), 3 months post-last treatment (C), and 6 months post-last treatment (D). (E–H) Untreated left hip at baseline (E), final treatment; T4 (F), 3 months post-last treatment (G), and 6 months post-last treatment (H).

**Figure 10.**
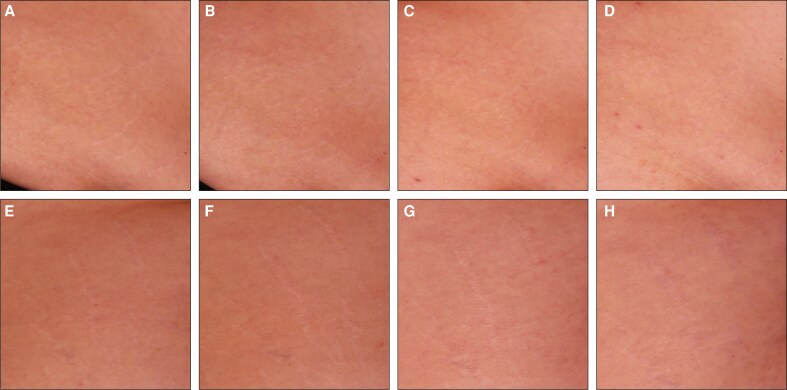
Site-matched treated and untreated striae albae on abdomen of 43-year-old female with FST IV. (A–D) Treated right abdomen at baseline (A), final treatment; T4 (B), 3 months post-last treatment (C), and 6 months post-last treatment (D). (E–H) Untreated left abdomen at baseline (E), final treatment; T4 (F), 3 months post-last treatment (G), and 6 months post-last treatment (H).

To complement the clinical assessments in Figures 1 and 2, standardized photographs of treated regions and corresponding untreated areas revealed visible improvements in treated striae at the final treatment visit (T4) and the 3- and 6-month follow-ups (Figures 3-10). Improvements were evident in both striae rubrae (Figures 3-6) and striae albae (Figures 7-10), with notable changes including reduced discoloration, smoother texture, and diminished lesion prominence relative to baseline. While some degree of variability in the extent of visible improvement was observed across skin types and individuals, a consistent pattern of greater improvement in treated compared to untreated regions was apparent across both striae subtypes (Figures 3-10). Mild changes were also observed in certain untreated regions, particularly in striae rubrae, which may reflect natural maturation over the 9-month observation period. While natural progression is an important consideration when assessing treatment outcomes, the visible differences in improvement between treated and untreated regions across subjects nonetheless support the aesthetic benefits of microneedling for both striae albae and striae rubrae (Figures 3-10).

In support, subject agreement with treatment effectiveness remained high across visits and lesion types. At the 6-month follow-up, 100% of participants with striae rubrae agreed that the microneedling treatments effectively improved the color, contour, texture, and overall appearance of their stretch marks (Table 3). Among those with striae albae, 95% reported agreement with most categories, with marginally lower agreement (89%) in texture/feel at study conclusion (Table 3). Participants who did not agree selected a neutral response (“neither agree nor disagree”), and no participants disagreed with any of the 4 statements at any timepoint. Quantitative distributions of subject satisfaction responses for each group are represented in Supplemental Figure 1.

**Table 3.** Percent Subject Agreement With Treatment Effectiveness Statements by Group and Visit

	Final treatment (T4)	3-month follow-up	6-month follow-up
Improved Color and Contour			
Striae Rubrae	100%	100%	100%
Striae Albae	100%	95%	95%
Improved Texture/Feel			
Striae Rubrae	87%	100%	100%
Striae Albae	100%	100%	89%
Reduced Size			
Striae Rubrae	87%	100%	100%
Striae Albae	95%	89%	95%
Improved Overall Appearance			
Striae Rubrae	87%	100%	100%
Striae Albae	95%	95%	95%

Those not in agreement were neutral (did not agree nor disagree).

**Figure 11.**
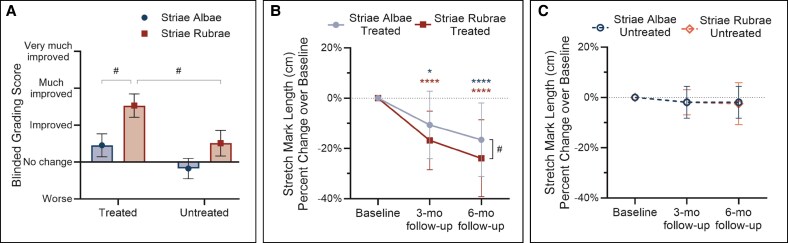
Quantitative assessments of treated vs untreated control sites at follow-up visits compared to baseline. (A) Blinded photo grading outcomes at 6-month follow-up for treated and untreated striae rubrae (red) and striae albae (blue). Shown are mean scores ± SEM based on a scale from −3 (much worse) to 3 (very much improved). Y-axis labels represent range of −1 (bottom) to 3 (top). *n* = 3 independent graders. (B, C) Percent change in longest stretch mark length (cm) over baseline at 3- and 6-month follow-up visits for treated groups (B) and untreated groups (C). Asterisks correspond to the respective stretch mark subtype by color and denote statistically significant improvement (*P < .05, ****P < .0001) over baseline. Number signs indicate significance between groups (^#^*P* < .05).

To substantiate our photographic observations and determine whether improvements in treated striae were attributable to the microneedling treatments rather than natural maturation alone, we performed quantitative analyses to more rigorously compare treated and untreated control sites. We designed a blinded evaluator grading system using a modified CGAIS scale in which paired baseline and 6-month follow-up photographs of all treated and site-matched untreated regions were randomly arranged and graded by 3 independent clinical experts who were blinded to treatment status and visit (Figure 11A). For each photo set, graders identified which image corresponded to baseline and which to the 6-month follow-up, then rated the degree of improvement over the image assigned to baseline using a 0-3 scale (0 = no change, 1 = improved, 2 = much improved, 3 = very much improved). Instances in which graders incorrectly assigned baseline/follow-up status yet rated improvement were adjusted to negative values, denoting deterioration rather than improvement.

Blinded photo grading using this modified scoring system demonstrated greater improvements in treated compared to untreated regions across both striae rubrae and striae albae, with the most pronounced benefit observed in striae rubrae (*P* < .01, Figure 11A). While mild improvements were detected in some untreated striae rubrae sites, likely reflecting natural maturation over study duration, the treated regions consistently achieved more favorable scores (Figure 11A).

Similarly, treated striae albae, though not significant, had consistently higher scores than their site-matched controls (Figure 11A). These more conservative grading scores may be explained by striae albae being less apparent in photographs than striae rubrae. However, blinded grading outcomes were corroborated by unblinded standard CGAIS photo grading for 3- and 6-month follow-up visits (Supplemental Figure 2), which likewise showed superior improvements in treated compared with untreated regions, including statistically significant (*P* < .001) differences between treated and untreated striae rubrae (Supplemental Figure 2A) and striae albae (Supplemental Figure 2B) at both timepoints. Taken together, these data support microneedling as the primary driver of the observed improvements as opposed to time-dependent changes alone.

To further substantiate our observations, we next compared changes in striae size, as measured by the length of the longest stretch mark in the designated treated and untreated control regions, to more objectively quantify changes in stretch mark appearance across timepoints (Figure 11B, C). In treated striae rubrae and albae, microneedling led to significant progressive reductions in striae length (Figure 11B), whereas the corresponding untreated regions exhibited minimal change across timepoints (Figure 11C). Despite the relatively large inter-individual variability reflected by wide standard deviations, the overall effects of the microneedling treatments remained robust, with treated regions showing statistically significant improvement over untreated controls at both follow-up visits (*P* < .001) (Figure 11B). These findings offer additional evidence to suggest that the aesthetic improvements presented herein extend beyond the effects of natural maturation alone.

In alignment with our earlier findings, both striae subtypes also showed continued reductions in length between 3- and 6-months posttreatment, further supporting the importance of this timeframe in the tissue remodeling process (Figure 11B). Moreover, reductions in stretch mark length were significantly greater in striae rubrae (23.9%) than striae albae (16.6%) at the 6-month follow-up (*P* < .05), again suggesting a potentially enhanced responsiveness in earlier-stage lesions (Figure 11B).

Taken together, these efficacy data underscore microneedling as an effective treatment modality for stretch marks across a range of skin types (FST I–VI), anatomical locations (abdomen, hips, shoulders), and stages of striae maturation, with the greatest improvements observed in striae rubrae.

### Safety and Tolerability

**Figure 12.**
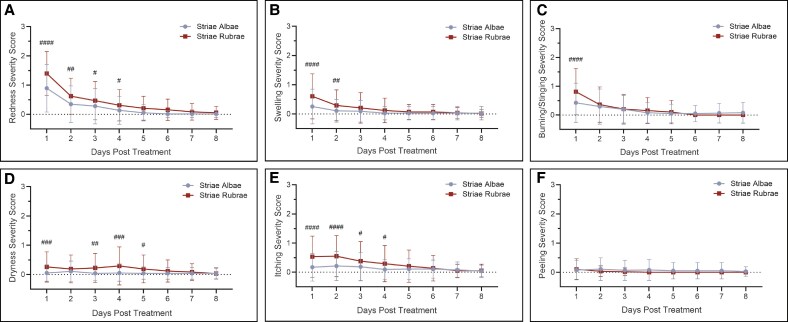
Subject-reported severity and resolution of common post-procedure symptoms over the first 8 days following microneedling treatment. (A–F) Daily mean ± SD symptom severity scores (based on a 4-point scale from 1 [none] to 4 [severe]) days 1-8 post-procedure for striae albae (blue; *n* = 19) and striae rubrae (red; *n* = 15) for redness (A), swelling (B), burning/stinging (C), dryness (D), itching (E), and peeling (F). Values represent pooled outcomes across all 4 microneedling treatments. Number signs indicate significance between groups (^#^*P* < .05, ^##^*P* < .01, ^###^*P* < .001, ^####^*P* < .0001).

Importantly, microneedling was well tolerated by all subjects. Responses to the procedure were among the typical reactions to microneedling and predominantly rated as mild by the study investigator (Table 4). The most common symptoms among study participants were mild erythema and itching, both generally mild, though nearly one-third of the striae rubrae group also experienced burning or stinging (Table 4). Similarly, subject-reported symptom severity was mostly scored as mild across symptoms, with scores being highest in the first 1-2 days posttreatment and gradually lessening with time (Figure 12A–F). Symptoms were resolved within eight days posttreatment for both striae subtypes (Figure 12A–F).

**Table 4.** Clinical Safety Grading Outcomes by Group

	Mild	Moderate	Severe
Erythema			
Striae Rubrae	32.2%	28.9%	3.3%
Striae Albae	43.9%	19.3%	0%
Edema			
Striae Rubrae	11.1%	0%	0%
Striae Albae	0.9%	0%	0%
Burning/Stinging			
Striae Rubrae	24.4%	5.6%	1.1%
Striae Albae	1.8%	0%	0%
Dryness			
Striae Rubrae	10%	0%	0%
Striae Albae	7.9%	0%	0%
Itching			
Striae Rubrae	16.7%	1.1%	1.1%
Striae Albae	17.5%	2.6%	0%
Scaling/Peeling			
Striae Rubrae	0%	0%	0%
Striae Albae	6.1%	0%	0%

Shown is the percentage of treatment regions that received mild, moderate, or severe grading scores based on investigator's assessment of post-microneedling safety parameters. Values represent the cumulative percentage across treatments.

It should be noted that striae rubrae regions did exhibit slightly higher rates of moderate or severe treatment responses compared to striae albae, particularly for erythema (28.9% vs 19.3% moderate) and burning/stinging (5.6% vs 0% moderate) based on clinical grading (Table 4). Likewise, the striae rubrae group reported consistently higher symptom severity scores in several parameters (eg, erythema, itchiness, dryness, burning) during the acute posttreatment period (days 1-4) than striae albae (Figure 12A–E). This may be expected due to the heightened activity and sensitivity of the striae during this early stage in development. These more pronounced responses in striae rubrae are important considerations from a clinical standpoint. However, it should be emphasized that all symptoms subsided within 7-8 days in both groups (Figure 12A–F), and none were escalated to adverse events.

## DISCUSSION

This prospective clinical study demonstrates that microneedling, when used on its own, can effectively and safely provide sustained improvement in the appearance of both striae rubrae and striae albae. These conclusions are supported by data from multiple assessment tools, including Manchester Scar Scale (MSS), Clinician's Global Aesthetic Improvement Scale (CGAIS), length measurements, safety grading, and patient-reported outcomes, and are further underscored by comparative outcomes between site-matched treated vs untreated regions. Treatment was well tolerated with consistent benefits across diverse skin types (FST I–VI) and anatomical locations, yielding high patient satisfaction with negligible adverse or undesired outcomes.

Our findings align with prior literature supporting microneedling's utility in dermal remodeling. Previous studies have reported improvements in skin texture, pigmentation, and laxity across various indications, including acne scars, surgical scars, and photoaged skin.^18-31^ However, few studies have isolated microneedling as a stand-alone therapy for stretch marks, and even fewer have compared efficacy based on striae type. By evaluating both subtypes over a 9-month evaluation period, including 6 months of follow-up, this study offers a more nuanced understanding of how microneedling performs across different stages of stretch mark development.

It should be highlighted that while both treatment groups showed significant improvement over the evaluation period, striae rubrae demonstrated greater overall responsiveness, particularly in visible texture, color, and length. While untreated striae rubrae also showed some improvement, albeit less than the treated sides, the heightened improvement seen in striae rubrae aligns with previous research on microneedling for early-stage surgical scars^31^ and may be attributed to the heightened vascular activity and collagen remodeling potential present during the acute inflammatory stage of striae development.^2,4^ These characteristics may make striae rubrae more responsive to stimulation with microneedling, though further research is needed to adequately elucidate the mechanisms behind this result.

Notably, the sustained improvements observed at both the 3- and 6-month follow-up visits suggest that skin remodeling continues well beyond the final treatment session. This is consistent with histologic studies showing continued collagen and elastin induction months after treatment.^18-20^ Patient-reported outcomes further underscore the clinical relevance of these findings, with nearly all participants noting improvements in the color, texture, and size of their stretch marks, consistent with MSS scores and striae length measurements.

Safety is a key consideration with any energy- or device-based treatment, especially for patients with darker skin tones who may be at higher risk for post-inflammatory hyperpigmentation (PIH).^32^ Importantly, this study found microneedling to be safe across all Fitzpatrick skin types, with most post-procedure reactions being mild and short-lived. Although patients with striae rubrae experienced slightly higher degrees of anticipated post-procedure reactions like erythema and stinging, these effects were transient and did not result in long-term complications.

From a clinical perspective, these findings are highly relevant. Stretch marks remain one of the most common yet challenging dermatologic and aesthetic complaints, particularly among postpartum women and adolescents.^2^ With limited success from topical therapies and greater costs and risks associated with energy-based options like laser treatments, microneedling stands out as a relatively cost-effective, low-risk, and well-tolerated alternative.^33^ Its ability to address both early- and late-stage lesions allows for versatility across widespread patient populations, and its favorable safety profile and minimally invasive approach make it particularly well suited for patients seeking natural-looking improvement with minimal downtime. Moreover, the findings presented herein can offer practical insight into treatment expectations and outcomes for patients with early- vs late-stage striae distensae.

### Limitations and Future Directions

While the results are compelling, limitations of the study include sample size and long-term follow-up. In particular, study participants were majority women (89.7%), and the anatomical locations of stretch mark regions were mainly limited to abdomen and hips, with 2 participants being treated on the shoulder/upper arm area. While we confirmed that study outcomes were not confounded by factors such as age, gender, FST or anatomical location, percent MSS improvement did trend downward with age (y = −0.3x + 55.08; *P* = .0720) (data not shown). Future studies with larger sample sizes would not only strengthen the statistical power of subgroup comparisons but may also reveal differential microneedling outcomes based on factors such as age or treatment location.

Moreover, the follow-up period in this study was limited to 6 months. While the study did not warrant a longer follow-up period with respect to safety, a longer-term evaluation period would be beneficial in establishing and understanding treatment durability effects. This is especially relevant considering we observed continued improvements between 3- and 6-months post-microneedling in several assessments, thus prompting the need for longer-term evaluations in future studies.

While the study included blinded photo grading and site-matched control comparisons based on CGAIS and stretch mark size, additional comparisons between treated and untreated sides may better account for natural progression/regression of stretch marks over time. Future randomized trials with untreated controls and comparisons to other modalities, such as fractional lasers, radiofrequency microneedling, or combination therapies, would illuminate the advantages/disadvantages of microneedling and help refine optimal treatment protocols. Histological studies and biomarker analyses may also shed light on the biological mechanisms driving differential responses between striae rubrae and albae, ultimately supporting the development of more personalized treatment approaches.

## CONCLUSIONS

Taken together, the safety and efficacy profiles presented herein both support and build upon prior studies looking at microneedling treatment for stretch marks. This clinical study not only affirms microneedling (using 2 different commercially available devices) as a safe and effective therapeutic modality for improving the cosmetic appearance of both striae rubrae and striae albae but also demonstrates better aesthetic outcomes with striae rubrae, suggesting that intervening early, while the lesions are still developing, (striae rubrae) can yield greater clinical outcomes compared to microneedling for post-maturation stretch marks (striae albae). To our knowledge, this is the first study to directly compare the clinical efficacy of microneedling for striae rubrae vs striae albae over an extended (6 month) follow-up period.

Overall, this study contributes to the growing body of evidence that supports microneedling as a valuable tool in aesthetic dermatology. Given its broad applicability, relatively low risk profile, and strong patient outcomes, microneedling should be considered a first-line treatment option, whether on its own or in combination with other treatments, in the management of striae distensae.

## Supplemental Material

This article contains supplemental material located online at https://doi.org/10.1093/asj/sjaf261.

## Supplementary Material

sjaf261_Supplementary_Data
