# Supplementary material for: A Comparative Study to Evaluate the Safety and Efficacy of Microneedling as a Stand-Alone Treatment for Striae Rubrae and Albae
Source: Aesthet Surg J. 2025 Dec 17;46(5):530–42. doi: 10.1093/asj/sjaf261 (PMC13064658; doi:10.1093/asj/sjaf261)
Supplement: sjaf261_Supplementary_Data [file sjaf261_supplementary_data.zip › Supplemental Table 1.docx]

| Supplemental Table 1. Treatment depth statistics by striae type and visit. *P*-values represent statistical comparisons between striae types at each monthly treatment visit (T1-T4) based on two-way ANOVA with Tukey’s multiple comparisons. | | | | | |
| --- | --- | --- | --- | --- | --- |
| Treatment Depth (mm) | **Striae Rubrae** | | **Striae Albae** | | *P*-value |
|  | Mean | SD | Mean | SD |  |
| T1 | 0.99 | 0.95 | 0.95 | 0.44 | 0.7585 |
| T2 | 1.19 | 1.17 | 1.17 | 0.40 | 0.8774 |
| T3 | 1.42 | 1.24 | 1.24 | 0.38 | 0.1816 |
| T4 | 1.45 | 1.34 | 1.34 | 0.38 | 0.4063 |
