# Supplementary figures and images for: A Comparative Study to Evaluate the Safety and Efficacy of Microneedling as a Stand-Alone Treatment for Striae Rubrae and Albae
Source: Aesthet Surg J. 2025 Dec 17;46(5):530–42. doi: 10.1093/asj/sjaf261 (PMC13064658; doi:10.1093/asj/sjaf261)

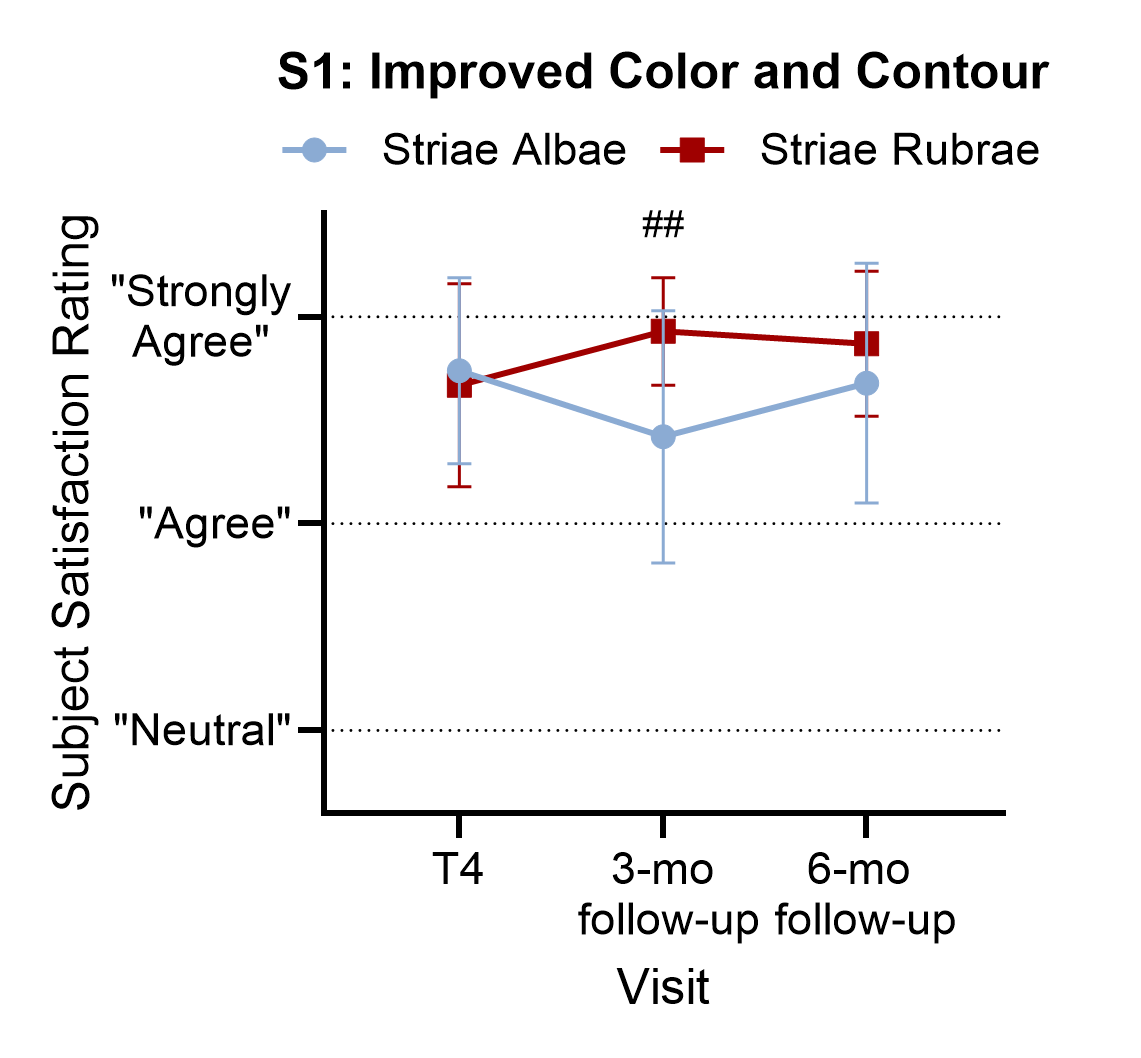

Supplement: sjaf261_Supplementary_Data [file sjaf261_supplementary_data.zip › Supplemental Figure 1 Part A.tif]

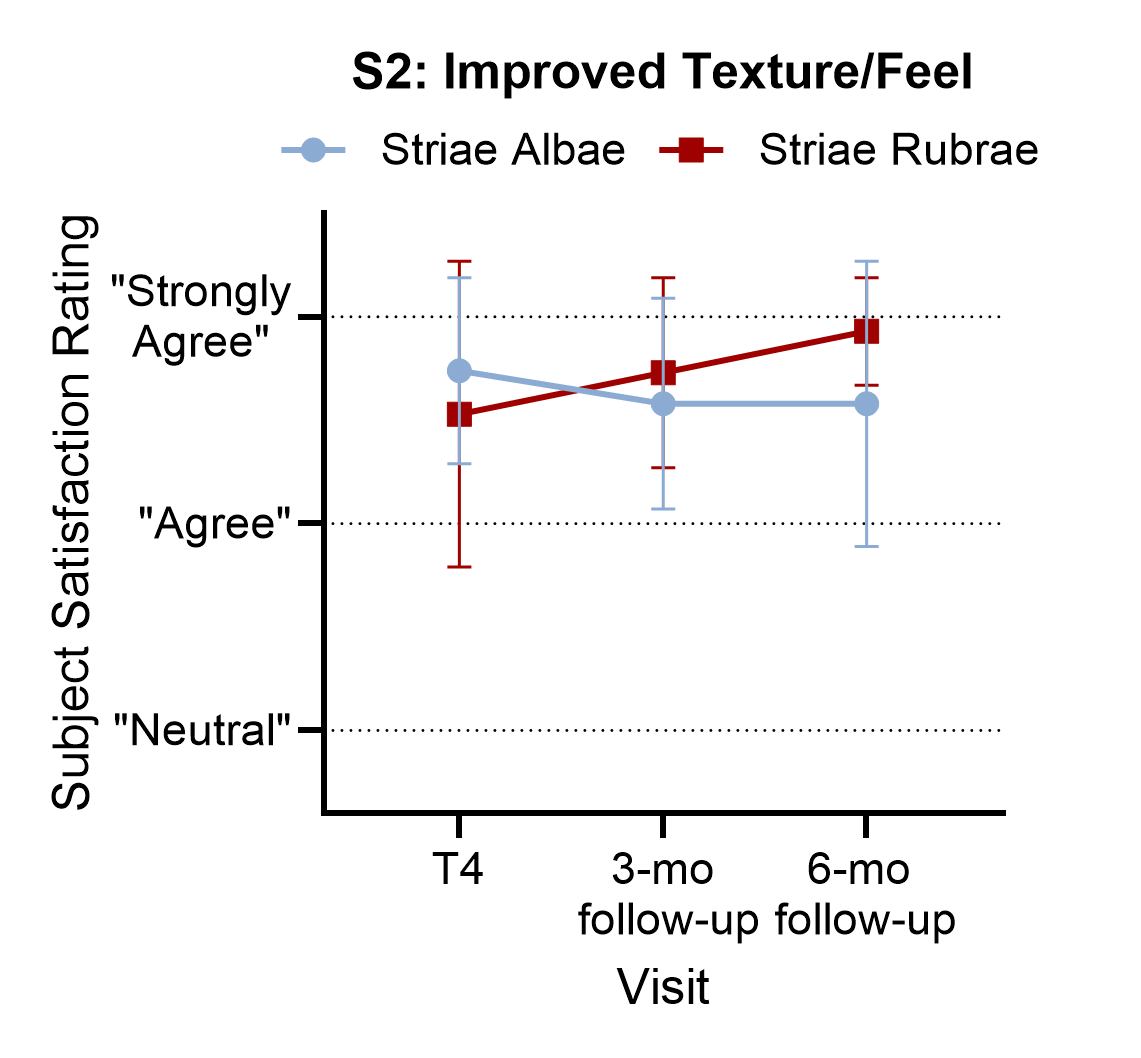

Supplement: sjaf261_Supplementary_Data [file sjaf261_supplementary_data.zip › Supplemental Figure 1 Part B.tif]

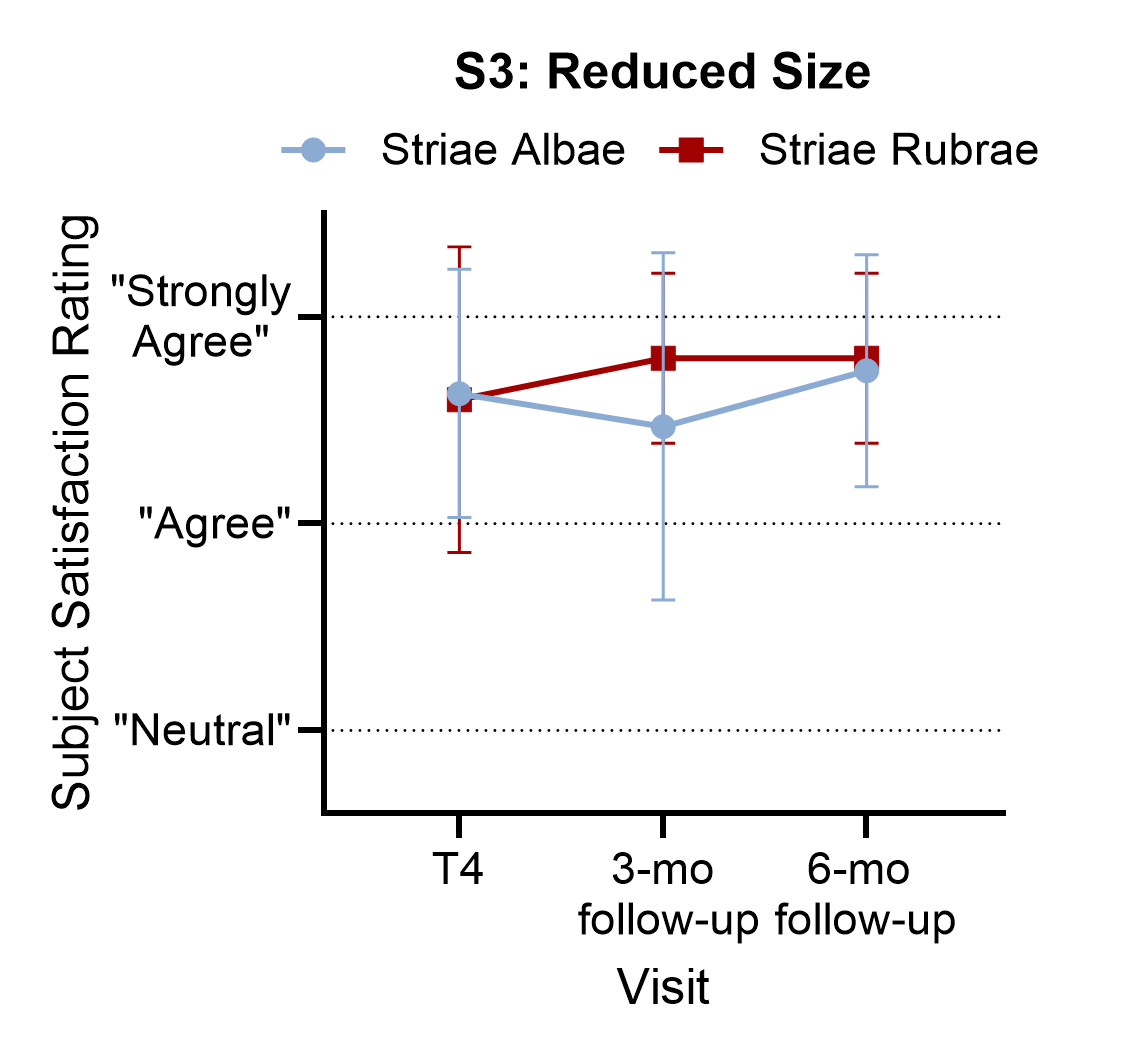

Supplement: sjaf261_Supplementary_Data [file sjaf261_supplementary_data.zip › Supplemental Figure 1 Part C.tif]

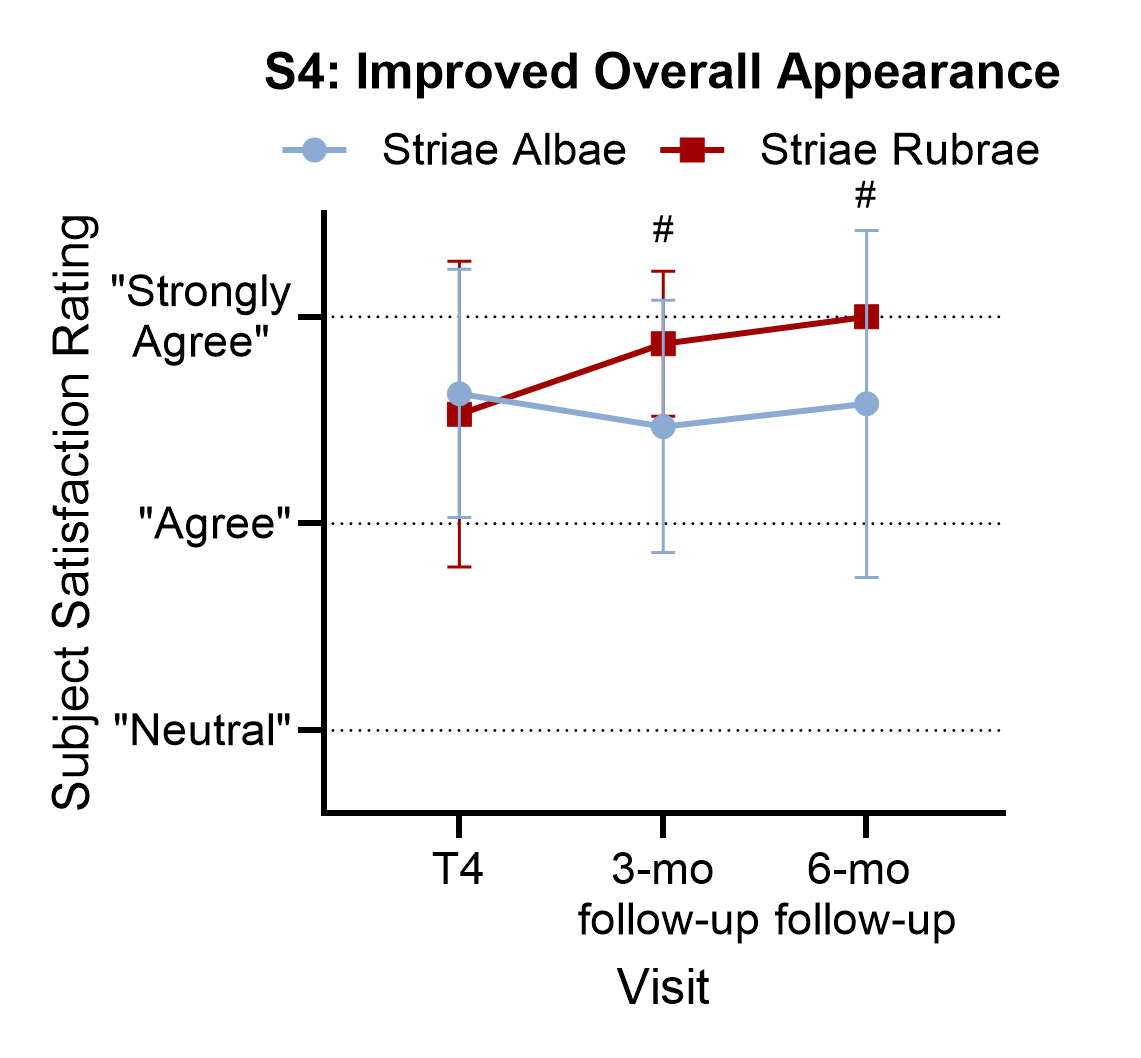

Supplement: sjaf261_Supplementary_Data [file sjaf261_supplementary_data.zip › Supplemental Figure 1 Part D.tif]

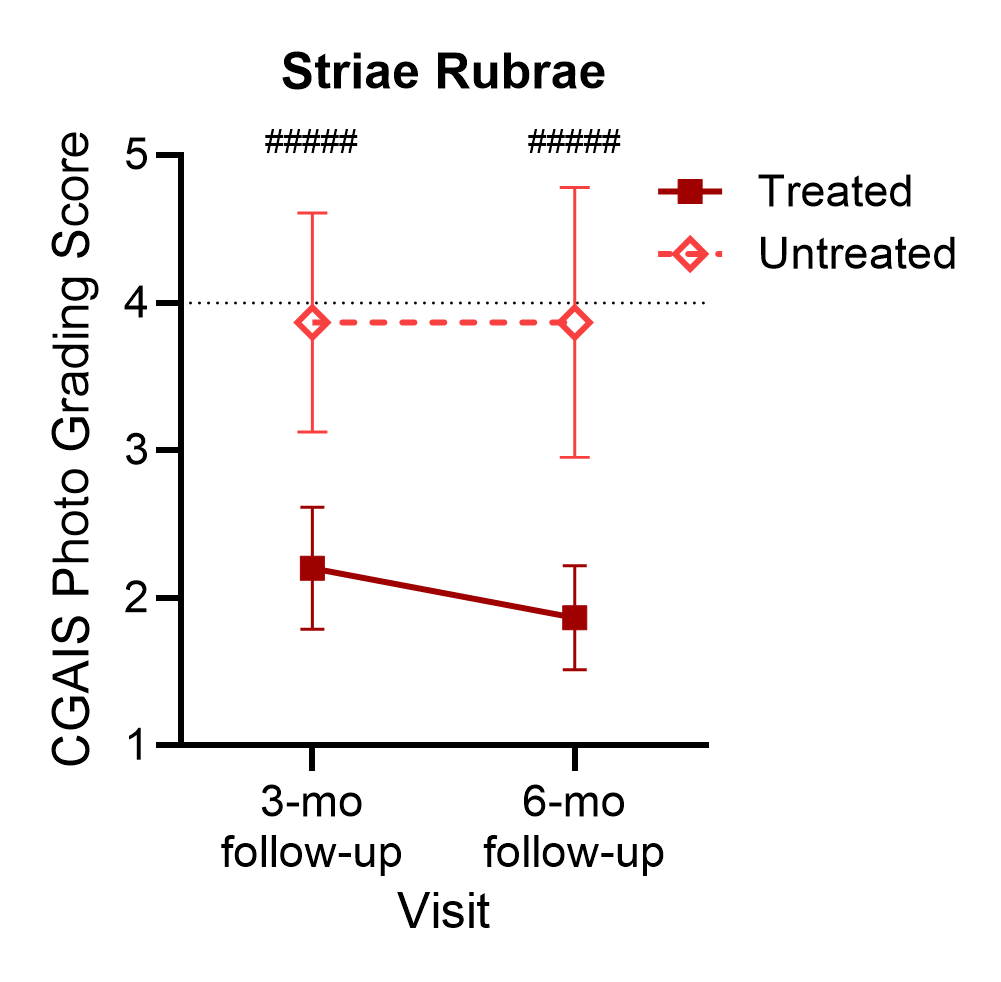

Supplement: sjaf261_Supplementary_Data [file sjaf261_supplementary_data.zip › Supplemental Figure 2 Part A.tif]

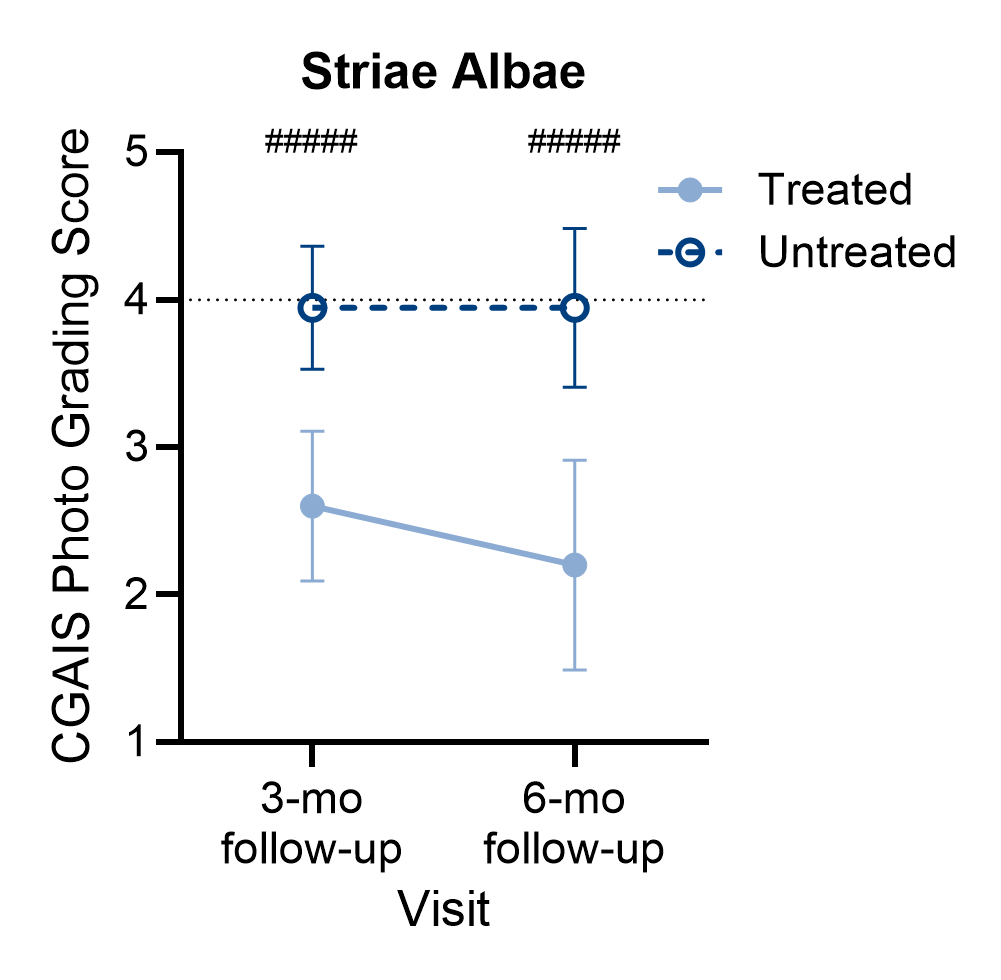

Supplement: sjaf261_Supplementary_Data [file sjaf261_supplementary_data.zip › Supplemental Figure 2 Part B.tif]
